# LEMONS – A Tool for the Identification of Splice Junctions in Transcriptomes of Organisms Lacking Reference Genomes

**DOI:** 10.1371/journal.pone.0143329

**Published:** 2015-11-25

**Authors:** Liron Levin, Dan Bar-Yaacov, Amos Bouskila, Michal Chorev, Liran Carmel, Dan Mishmar

**Affiliations:** 1 Department of Life Sciences, Ben Gurion University of the Negev, Beer Sheva, 8410501, Israel; 2 Department of Genetics, The Alexander Silberman Institute of Life Sciences, The Hebrew University of Jerusalem, Jerusalem, 91904, Israel; 3 School of Computer Science and Engineering, The Hebrew University of Jerusalem, Jerusalem, 91904, Israel; University of Connecticut, UNITED STATES

## Abstract

RNA-seq is becoming a preferred tool for genomics studies of model and non-model organisms. However, DNA-based analysis of organisms lacking sequenced genomes cannot rely on RNA-seq data alone to isolate most genes of interest, as DNA codes both exons and introns. With this in mind, we designed a novel tool, LEMONS, that exploits the evolutionary conservation of both exon/intron boundary positions and splice junction recognition signals to produce high throughput splice-junction predictions in the absence of a reference genome. When tested on multiple annotated vertebrate mRNA data, LEMONS accurately identified 87% (average) of the splice-junctions. LEMONS was then applied to our updated Mediterranean chameleon transcriptome, which lacks a reference genome, and predicted a total of 90,820 exon-exon junctions. We experimentally verified these splice-junction predictions by amplifying and sequencing twenty randomly selected genes from chameleon DNA templates. Exons and introns were detected in 19 of 20 of the positions predicted by LEMONS. To the best of our knowledge, LEMONS is currently the only experimentally verified tool that can accurately predict splice-junctions in organisms that lack a reference genome.

## Introduction

Large scale analyses of multiple genes are currently possible at continuously decreasing costs due to advances in massively parallel sequencing technologies, such as whole genome re-sequencing [1], exome sequencing [2], and RNA-seq [3], and increased computational efficiency, especially in *de novo* assembly techniques [4]. For organisms lacking a fully sequenced reference genome, RNA-seq emerges as the method of choice, avoiding the computational burden of *de novo* genome assembly. RNA-seq provides valuable information on gene annotation and genome-wide expression differences among tissues and individuals while enabling identification of alternatively spliced variants [5].

A key property of many eukaryotic genes, especially in vertebrates, is their organization into multiple exons, which are divided by introns. Introns are removed by splicing, thus leading to intron-less mature transcripts. This fundamental property hampers the direct use of RNA sequences as references for DNA-based studies, especially in organisms lacking a reference genome. Additionally, obtaining RNA-seq data (i.e., transcriptomes) remains costly and, therefore, only few recent efforts have been made by molecular evolutionists and ecologists to perform population genomics studies based solely on RNA-seq data [6–8]. Thus, to analyze multiple samples, such researchers, who often study species with little or no genomic information, prefer using DNA for their purposes. This especially applies when large sample sizes are required, as in the case of population studies or experimental investigation of the evolution of non-model organisms. Such an approach, however, restricts analysis to either highly studied sequences, such as the mitochondrial genome, to a limited number of highly conserved nuclear DNA loci, or when prior knowledge of the genomic reference, such as when Amplified Fragment Length Polymorphism (AFLP) is involved, is not required. Whereas tools exist for the study of population dynamics in such scenarios, the unbiased identification of genes that are important for processes such as adaptation, hybrid breakdown or speciation require data at the genomics level from multiple samples of a studied organism. Therefore, there is need for a tool that enables identification of exon-exon junctions in RNA sequences. Such a tool would facilitate the subsequent isolation of genes of interest in DNA samples.

The vast majority of currently available splice-junction prediction tools identifies exon-exon boundaries in mRNA sequences by comparing RNA to the underlying DNA sequence of the same organism [9–15], thus rendering them inapplicable for organisms that lack a reference genome. Twenty years ago, efforts were made to predict splice-junctions in RNA sequences without a reference genome [16]. These efforts generated an early tool that was limited to human sequences and that preceded the ‘omics’ era and, therefore, could not be used for analyzing complex whole-genome RNA-seq data. Moreover, the exonic information content assessing splice-junctions was very low, severely limiting the usefulness of this tool. More recently, CEPiNS, a bioinformatics tool designed to identify exon-exon boundaries in RNA sequences regardless of the availability of a DNA reference sequence, was created [17]. The designers of this tool reasoned that exon/intron junctions are highly conserved, relative to the coding sequence [18–24]. However, levels of accuracy were not reported for CEPiNS, it did not employ a motif search, it used only a single reference genome and it was not verified experimentally.

Here, we present LEMONS, a user-friendly software that predicts exon-exon junctions along mRNA sequences even in the absence of a reference genome. LEMONS achieves high precision by simultaneous consulting multiple reference genomes and by searching for splice site recognition motifs. We tested the efficacy of LEMONS in predicting splice-junctions in vertebrates, and demonstrated the power of this tool by experimentally verifying a subset of its predictions for the Mediterranean chameleon, an organism that lacks a reference genome.

## Materials and Methods

### Design of LEMONS

**Fig 1:**
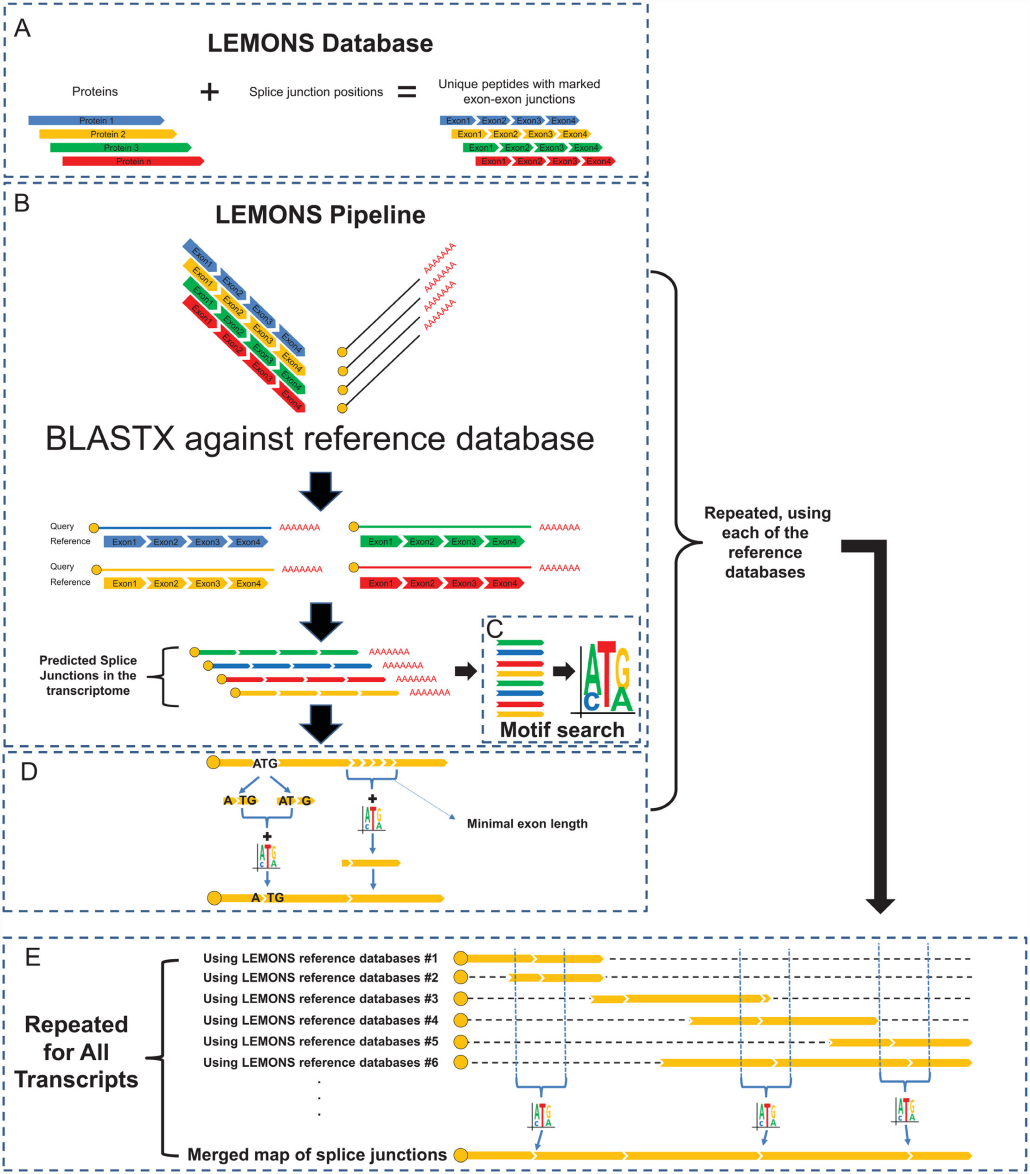
Flow chart of the steps performed by LEMONS. (A) LEMONS default and primary database taken from UCSC Genome Browser and HG19 encompasses all non-redundant human RefSeq proteins, together with their known splice-junctions location. Arrowhead-like gaps correspond to splice-junctions. (B) LEMONS employs BLASTX pairwise alignment to compare each of the identified transcripts to their orthologous proteins (as compared to the reference database) and predicts splice-junctions based on the conserved gene structure. (C) LEMONS uses all predicted exons that do not split codons to establish the 3' motif of the exon. (D) The identified motif assists in choosing between adjacent potential splice-junctions and between the two potential splice-junctions that split codons. (E) Using more than one reference database enhances the accuracy of splice-junction prediction (again, while implementing a motif search).

LEMONS was written in PYTHON (http://www.python.org/) and converted into a Windows executable program using the Py2exe extension package (http://www.py2exe.org/). The executable files, source code, graphical user interface (GUI) and a Linux version of the program, as well as a user manual, are available at: http://dx.doi.org/10.6084/m9.figshare.1599765. The LEMONS database includes six well-annotated vertebrate genomes/proteomes as reference genomes, namely those from *Homo sapiens*, *Mus musculus*, *Gallus gallus*, *Anolis carolinensis*, *Xenopus tropicalis* and *Danio rerio*. LEMONS also includes databases for the three non-vertebrate model species *Arabidopsis thaliana*, *Caenorhabditis elegans* and *Drosophila melanogaster*. The main reference was defined as the human genome, constructed using three resources: (1) human RefSeq proteins (http://www.ncbi.nlm.nih.gov/), (2) their genomic location and exon distribution along the hg19 assembly (UCSC Genome Browser http://genome.ucsc.edu/), and (3) the human hg19 assembled genome (GenBank, GCA_000001405.1). LEMONS uses BLASTX (BLAST 2.2.28+ package [25]) to align assembled RNA-seq data or mRNA sequences of interest (query) against the set of reference peptides, with LEMONS choosing the best match, i.e., the highest BLAST score. This allows for identification of the best candidate orthologous transcripts of the reference proteins. LEMONS then predicts splice junction sites based on sequence similarity and the conservation of exon boundaries (Fig 1). Users can either select their preferred reference out of a set of six well-annotated genomes found in the LEMONS database, or simultaneously consult all available reference genomes (see below). LEMONS incorporates a built-in database generator that enables users to generate their own reference genome using publicly available genomes and genome annotation.

### Assessing the precision of predictions made by LEMONS

To test LEMONS, we first applied the program to 46 fully sequenced vertebrate genomes, using the latest genome assemblies and the corresponding sets of non-redundant predicted peptides, together with their genomic location and exon mappings, as extracted from the UCSC Genome Browser (genome.ucsc.edu/). To ensure that only well annotated transcripts were considered, we compared dataset A, containing the translated transcript database of each tested organism, and database B, containing the corresponding sequences of all genes extracted from the genome, in which only annotated exons were merged and translated. Only transcripts that were identical in the two datasets were used for subsequent analyses. We, furthermore, calculated the ratio between the number of such transcripts and the total number of transcripts (including non-identical ones) in the tested species, using the formula: $\frac{Number\;of\;A(i)\;=\;B(i)}{Total\;number\;oftranscripts\;}$, where A(i) and B(i) refer to matched transcripts from each of the two datasets, respectively. The resulting values were referred to as the ‘Annotation Quality’ (S1 Fig) of the tested genomes.

### Screening for splice site recognition sequences and the simultaneous use of multiple reference databases

Since LEMONS utilizes protein-based databases, the exact localization of splice-junctins could be directly computed only for those that do not split codons (i.e. the splice-junction could be positioned either before or after the codon). To address this caveat, we addressed all predicted splice-junctions that do not split codons (according to the LEMONS reference database) to identify a conserved motif at the 3’ end of exons [26]. Next, we sorted and graded the most probable motif among the predicted splice-junctions within split codons, based on sequence similarity to the identified motif (Fig 1). To further improve exon-exon boundary prediction, LEMONS employs a minimal exon length threshold. To compute this threshold, as well as the length of the splice site recognition motif, we used all six reference databases (i.e. *H*. *sapiens*, *M*. *musculus*, *G*. *gallus*, *A*. *carolinensis*, *X*. *tropicalis* and *D*. *rerio*), splice site recognition motif lengths up to 5 bp, and minimal exon lengths up to 20 bp (S2 Fig). We found that the best balance between sensitivity and precision (i.e., the ratio between precision gains to sensitivity loss) was achieved when the splice site recognition motif was reduced to a single base, and a minimum exon length of 10 bp was employed. Although longer recognition motifs are usually expected, a single base carries sufficient information for this task. Hence, these parameters were set as the default values, but can be modified by the user.

### Comparing the performances of LEMONS and CEPiNS

All comparisons were performed using the same machine (desktop computer containing an Intel i7 4770 CPU core with 16GB RAM and a 64bit operating system). In running CEPiNS, we excluded the filter for alternative splicing in the input sequences, as this feature led the program to crash with three of the five tested species.

### Transcriptome sequencing and assembly of the common Mediterranean chameleon

Blood was drawn (500 μl) from the tail of a single wild-caught Mediterranean chameleon during a night expedition in the north of Israel (UTM coordinates: 712957.26 E, 3635695.91 N, public area outside of Yodfat) using a sterile standard 1 ml syringe. The chameleon was released alive on site right after blood sample collection. All sampling procedures and experimental manipulations were approved as part of obtaining the field permit from the Israel Nature and Park Authority (permit 2013/40003), as required by the university committee for the ethical care and use of animals in experiments (IACUC number IL-18-03-2012). Total RNA was extracted from white blood cells using a Perfect pure RNA kit (5 Prime). RNA concentration was estimated using a nano-drop apparatus (NanoDrop Technologies). To further assure RNA quality, rRNA bands were visualized on 1% agarose. A library for massive parallel sequencing was prepared with a TruSeq RNA Kit (Illumina) according to the manufacturer’s protocol. RNA-library sequence reads (101 bp, paired ends) were generated with an Illumina HiSeq 2000 apparatus (Technion Genome Center, Haifa, Israel). The transcriptome was then *de novo* assembled using CLC-Bio Genomic workbench 6.01. The assembly resulted in 83,519 contigs longer than 200 bp (Table 1). The data can be accessed in Sequence Archive Reads (http://www.ncbi.nlm.nih.gov/sra), accession number SRP029972. We stress that LEMONS input comprises FASTA files. The user can assemble transcriptomes from their organisms of choice using any available assembly program to generate FASTA files. FASTA files generated by traditional Sanger sequencing can also be used.

**Table 1:** Summary statistics following analysis of the new version of the chameleon transcriptome (TransCham v2.0).

Category	Quantity
lower 25% of contig length (bp)	451
Median contig length (bp)	1,306
Upper 25% percentile contig length (bp)	3,165
Minimum contig length (bp)	200
Maximum contig length (bp)	23,487
Average contig length (bp)	751
Total number of contigs	83,519

### PCR amplification and sequencing

PCR amplifications of each studied gene were performed in a 25 μl volume (see S1 Table for the list of primers used and genes amplified). The genes analyzed were of varying transcript length, contained different number of exons and were found at various chromosomal locations. Reaction conditions are summarized in S2 Table. All reaction products were stored at -20°C until use. PCR products were visualized on an EtBr-stained 1% agarose gel, purified using Wizard SV Gel and PCR Clean-up systems (Promega) following the manufacturer’s protocol and sequenced (ABI 3100, BGU sequencing facility) using either one or two of the amplification primers, except for KIA0020. Sequence analysis was performed using Sequencher 4.9 (GeneCodes) and MEGA 5 [27].

## Results

### LEMONS—Localizing exon-exon boundaries in mRNAs of organisms with no sequenced reference genome

In the present report, we have created LEMONS, a user-friendly computational tool that identifies splice-junctions in transcriptome/multiple mRNA sequences while avoiding the need for a reference genome (Fig 1). LEMONS allows subsequent design of primers for PCR amplification within gene exons, thus facilitating subsequent DNA-based studies, especially in under-studied vertebrates. Known and mapped splice-junctions within the database provided references for the identification of putative splice-junctions in the orthologous mRNA sequences of the studied organism. To reduce the false discovery rate, analysis was restricted by default only to sequences with high similarity to human protein sequences (e-value < 1.0 X 10^−5^, see below). The output of LEMONS lists predicted splice-junctions within the query nucleotide sequence.

### LEMONS predicts splice junctions with high precision

To assess the precision of splice-junction predictions by LEMONS, we used publically available whole genome sequences from 46 vertebrates. Initially, we focused on humans and five model genomes representing commonly used model species belonging to five vertebrate classes with well annotated genomes (*Mammalia—M*. *musculus* (house mouse), *Aves—G*. *gallus* (chicken), *Reptilia—A*. *carolinensis* (green anole), *Amphibia—X*. *(Silurana) tropicalis* (western clawed frog) and *Actinopterygii—D*. *rerio* (zebrafish)).

**Fig 2:**
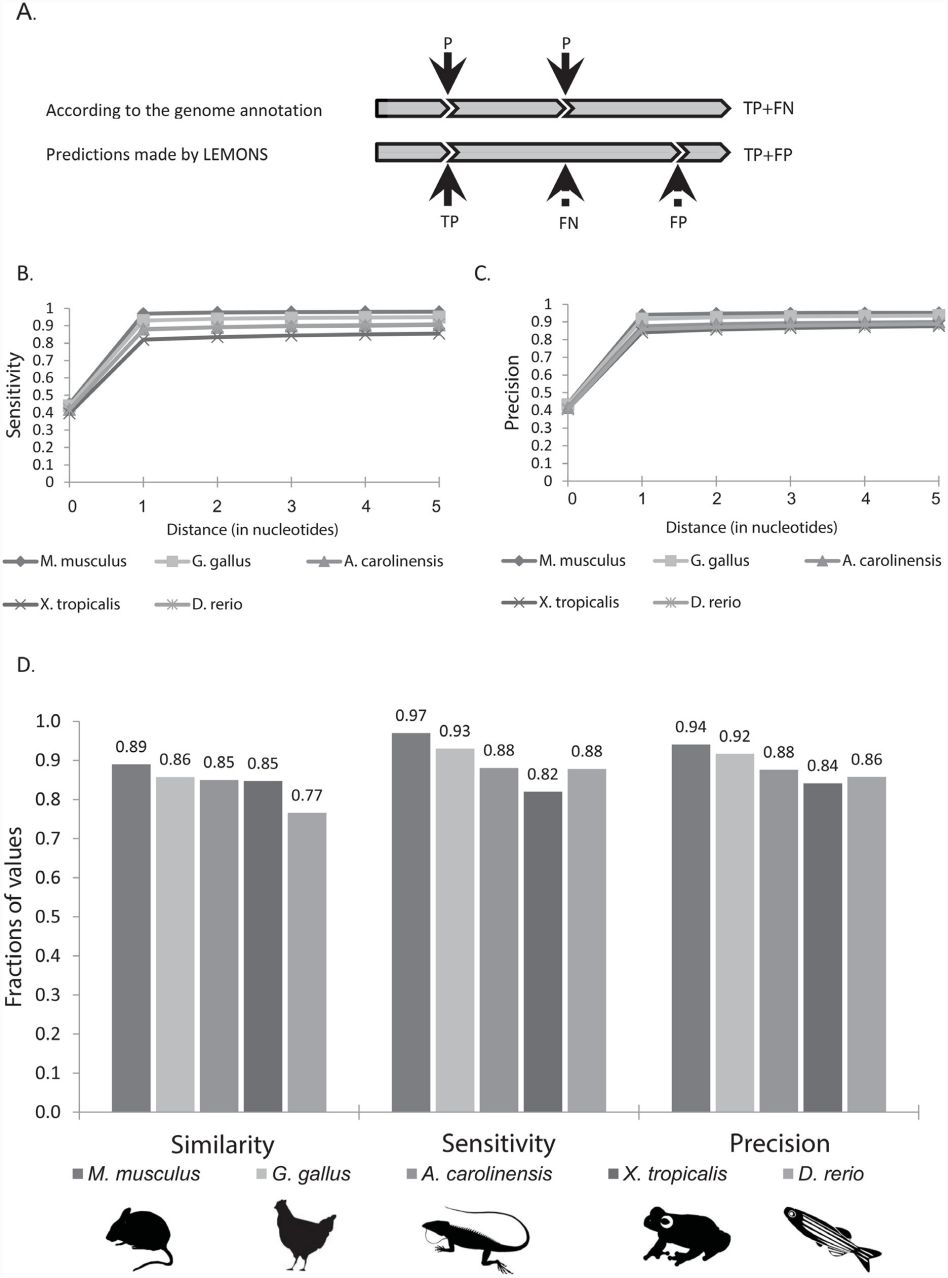
LEMONS sensitivity and precision assessment. (A) Demonstration of the different splice-junction predictions made by LEMONS and their occurrence in the examined organism’s coding regions, according to genome annotation. P—"true" splice-junction; TP (true positive)–correct identification of splice-junction by LEMONS; FN -false negative; FP—false positive splice-junctions. TP+FN (true positive + false negative)–total number of true splice-junctions in the examined organism, according to genome annotation; TP+FP (true positive + false positive)–total number of splice-junctions predicted by LEMONS; (B-C) LEMONS-based identification of splice-junctions. Our analysis accounted for the distance (in nucleotides) between the splice-junction predicted by LEMONS and the true splice-junction. The analysis presented is of five species: *M*. *musculus*, *G*. *gallus*, *A*. *carolinensis*, *X*. *tropicalis and D*. *rerio*. (D) Comparison of LEMONS similarity, sensitivity and precision for the five species tested. For absolute numbers, see S3 Table.

To determine the success rate of predictions made by LEMONS for these five organisms, we marked all known splice-junction positions. These were considered ‘true’ splice-junctions (P). These positions were compared to LEMONS-predicted splice-junctions, marked as TP and FP, representing true positive and false positive sites, respectively (Fig 2A). The sum of true positive and false negative predicted splice-junctions was marked as TP+FN. We found that LEMONS predicted splice-junctions up to a single nucleotide away from most (>82%) of the true splice-junctions in all five tested species (Fig 2B and 2C). Therefore, in subsequent analyses, identification of splice-junctions was considered correct only if the predicted site was no more than a single nucleotide way from the true splice-junction (TP). To define the sensitivity (i.e. recall) of our predictions, we next calculated the ratio between the number of correctly predicted and known splice-junctions in the entire set of human orthologs for each tested species ($\frac{TP}{TP+FN}$) (Fig 2D, S3 Table). Finally, we calculated the precision of LEMONS as the ratio between the number of TPs and the total number of splice-junctions predicted by LEMONS in the entire set of human orthologs for each tested species ($\frac{TP}{TP+FP}$).

To determine orthology relationships, we tested three different e-values (1.0 X 10^−5^, 1.0 X 10^−10^, and 1.0 X 10^−50^). The best results were obtained for an e-value of 1.0 X 10^−5^. At this threshold, LEMONS retained high precision and presented sensitivity values while attaining the highest fraction of total analyzed nucleotides in each of the five species considered. Specifically, there was, on average, a 5% increase in the similarity (i.e., the fraction of nucleotides analyzed $=\;\frac{Orthologous\;sequences\;length\;}{Total\;sequences\;length}$), yet only a 1% decrease in the precision and recall results using these parameters (S3 Fig). Consequently, we used the e-value 1.0 X 10^−5^ in analyzing the remaining 41 vertebrate species and in all subsequent analyses (S1 Fig). This value was set as default in LEMONS, although users can choose to use different values.

Next, we used the human genome reference as default database to identify orthologs. The mean similarity level of identified human orthologs (e-value < 1.0 X 10^−5^) was 89% in *M*. *musculus*, 86% in *G*. *gallus*, 85% in *A*. *carolinensis*, 85% in *X*. *tropicalis* and 77% in *D*. *rerio* (Fig 2D, S3 Table). LEMONS analysis revealed high levels of correct splice site predictions (sensitivity), namely 97% for *M*. *musculus*, 93% for *G*. *gallus*, 88% for *A*. *carolinensis*, 82% for *X*. *tropicalis*, and 88% for the *D*.*rerio*. The precision achieved by LEMONS ranged between 84–94% for the tested species. At the same time, the false discovery rate ($\frac{FP}{TP+FP}$) ranged from 6% to 16% (Fig 2D, S3 Table). Assignment of some of the false positive splice-junctions could have resulted from differences in the splice pattern in the tested species (e.g., exon skipping or inclusion). Such junctions may be known in human yet are not present in the tested species.

Using the same set of parameters as employed for analysis of the five species considered above, we examined 41 additional vertebrate species with publically available fully sequenced and annotated genomes and the corresponding mRNA databases (S1 Fig). In these analyses, we used the human genome as a reference, and found average sensitivity, precision and similarity values of 83%, 88% and 89%, respectively (medians of 88%, 88% and 90%, respectively). Notably, some of these 41 vertebrate genomes displayed relatively low sensitivity values (S1 Fig), presumably due to low genome ‘Annotation Quality’ (see Material and Methods and S1 Fig).

### A splice site recognition motif search and the simultaneous use of more than one reference genome increase the fraction of nucleotides analyzed

**Fig 3:**
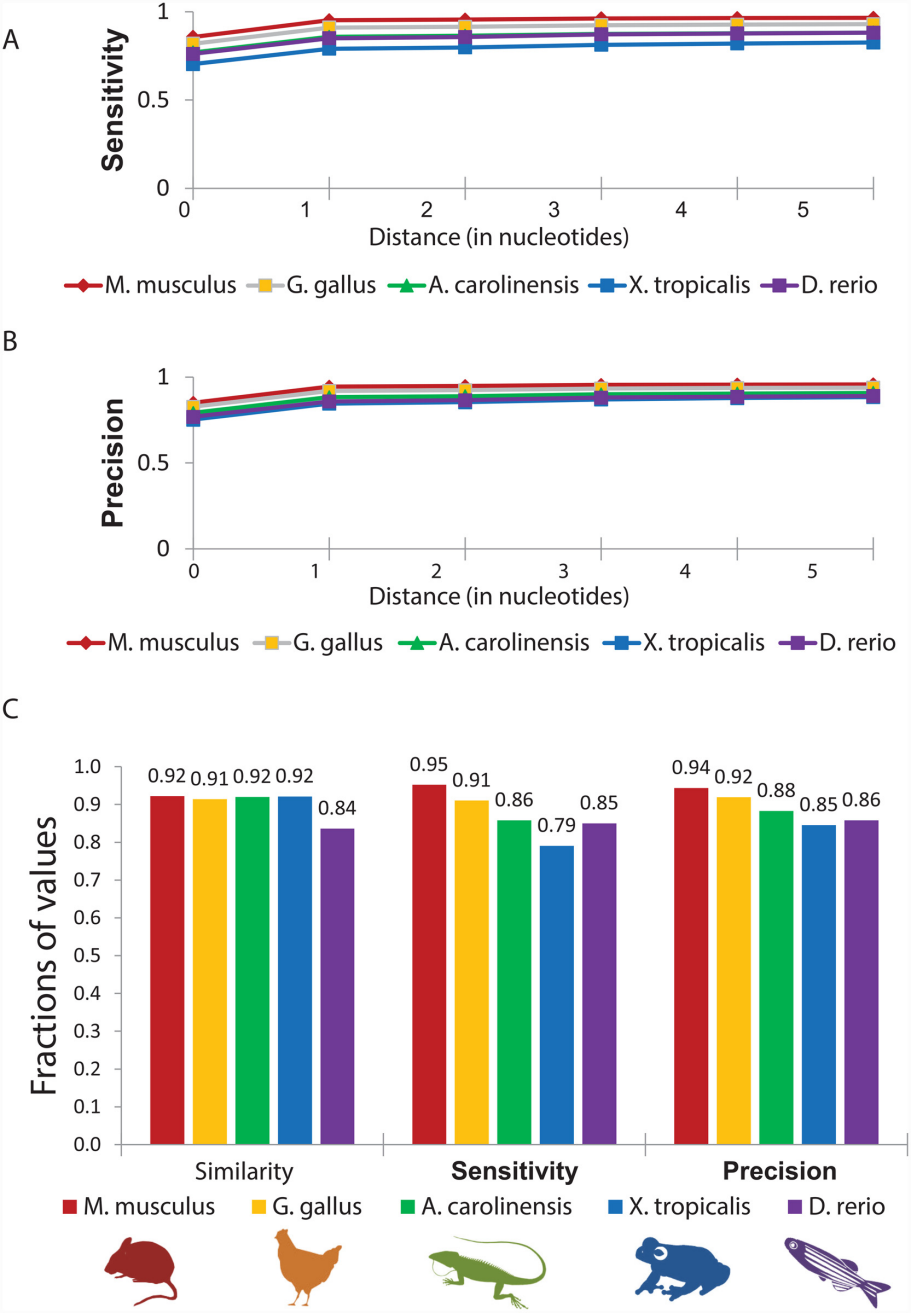
LEMONS sensitivity and precision assessment using a motif search and multiple reference databases. (A-B) Identification of splice-junctions by LEMONS. Our analysis accounted for the distance (in nucleotides) between splice-junctions predicted by LEMONS and the true splice junctions. (C) Comparison of LEMONS similarity, sensitivity and precision plotted for the five species tested. The analysis was performed using five databases, including the human and four of the model organisms (excluding the tested organism). The nomenclature used is as in the legend to Fig 2.

Using protein databases for our analysis likely reduced the precision of splice-junction identification, since amino acid sequences represent nucleotide codons, whereas splicing occurs at the single nucleotide level. Indeed, as mentioned above, identification of splice-junctions was considered correct if the predicted site was within a single nucleotide from the true splice-junction. We, therefore, sought to improve the precision of LEMONS predictions by applying two additional features, namely a splice-junction recognition motif search and the simultaneous use of more than one reference database. To assess the contribution of each feature, genomes of the five available model organisms (i.e. *M*. *musculus*, *G*. *gallus*, *A*. *carolinensis*, *X*. *tropicalis* and *D*. *rerio*) were considered by two types of analysis. In the first, results obtained while using only one reference genome (human) and without a splice site recognition motif search were examined (Fig 2). In the second, results obtained while simultaneously using five reference genomes, including the human genome and those of four of the model organisms (excluding that of the tested organism) were collected. Such analysis was supplemented by a splice site recognition motif search (Fig 3). Comparison of the two analyses revealed that inclusion of the motif search and simultaneous use of more than one reference database resulted in an average increase in similarity to 90%, all the while retaining the level of false discovery rate and increasing the false negative rate by only 3% on average to 13% (Figs 3C and 2D). Moreover, inclusion of the motif search improved the proportion of accurately predicted splice-junctions (i.e., where the distance between predicted and true splice-junctions equals zero) from an average sensitivity of 42% to 78%, and from an average precision of 42% to 80% (Figs 2B, 2C, 3A and 3B). We thus conclude that the simultaneous use of several reference databases in combination with performance of the motif search improved LEMONS precision.

### LEMONS outperforms CEPiNS

**Fig 4:**
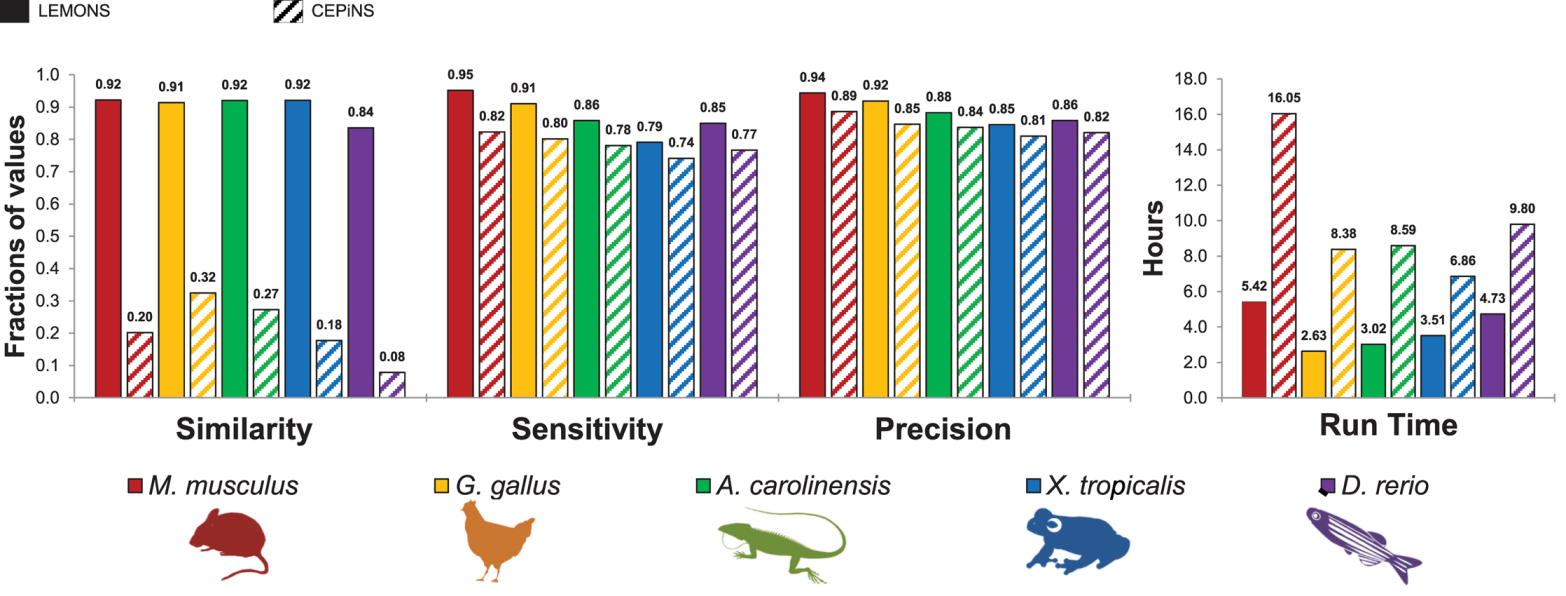
Comparing the performances of LEMONS and CEPiNS. LEMONS performance in terms of similarity, sensitivity and precision are as indicated in Fig 3. CEPiNS performance was evaluated using a human database (the same Refseq protein sequences as used in the LEMONS human database, including their genomic sequences). The same RNA sequences were used as input in both analyses. CEPiNS evaluation was performed using default settings, excluding the filter for alternative splicing in the input sequences (see Materials and Methods).

Hasan and Wheat previously described CEPiNS, a tool that like LEMONS, exploited gene structure conservation to predict exon-exon junctions in RNA sequences [17]. Two major differences, however, exist between CEPiNS and LEMONS. Firstly, unlike LEMONS that relies on multiple reference databases, CEPiNS uses a single reference (human), and does not conduct any motif search at all. Secondly, unlike LEMONS, which requires only the transcripts of the tested organism as an input file, CEPiNS requires its users to create their own reference database that includes the cDNA and reference genomic sequences of the relevant organism. In comparing the performance of LEMONS and CEPiNS in identifying exon-exon junctions while analyzing RNAs of *M*. *musculus*, *G*. *gallus*, *A*. *carolinensis*, *X*. *tropicalis* and *D*. *rerio*, we found that LEMONS outperformed CEPiNS in every test for each parameter (Fig 4). Strikingly, CEPiNS was able identify splice-junctions in only 32% of the analyzed transcripts, as compared to the more than 84% identified by LEMONS. We, therefore, concluded that LEMONS is currently the most efficient tool available for the identification of exon-exon junctions in RNAs, regardless of the availability of a reference genome sequence from the same organism.

### Sequencing and *de novo* assembly of the chameleon transcriptome

Recently, we sequenced and *de novo* assembled the first chameleon transcriptome [28]. For the sake of the current analysis, we aimed at increasing contig lengths. Hence, we sequenced and *de novo* assembled poly A-enriched mRNA extracts from white blood cells isolated from a wild-captured chameleon (*Chamaeleo chamaeleon recticrista*). Our *de novo* assembly resulted in a new version of the chameleon transcriptome (TransCham V2.0, available at: http://lifeserv.bgu.ac.il/wb/dmishmar/pages/lemons.php) encompassing 83,519 contigs longer than 200 bp with an average length of 751 bp and a median length of 1306 bp (see Table 1 for summary statistics). TransCham V2.0 was subsequently subjected to LEMONS analysis.

### Splice-junction predictions by LEMONS enabled successful DNA-based amplification and sequencing of exons and introns from the common Mediterranean chameleon genome

**Fig 5:**
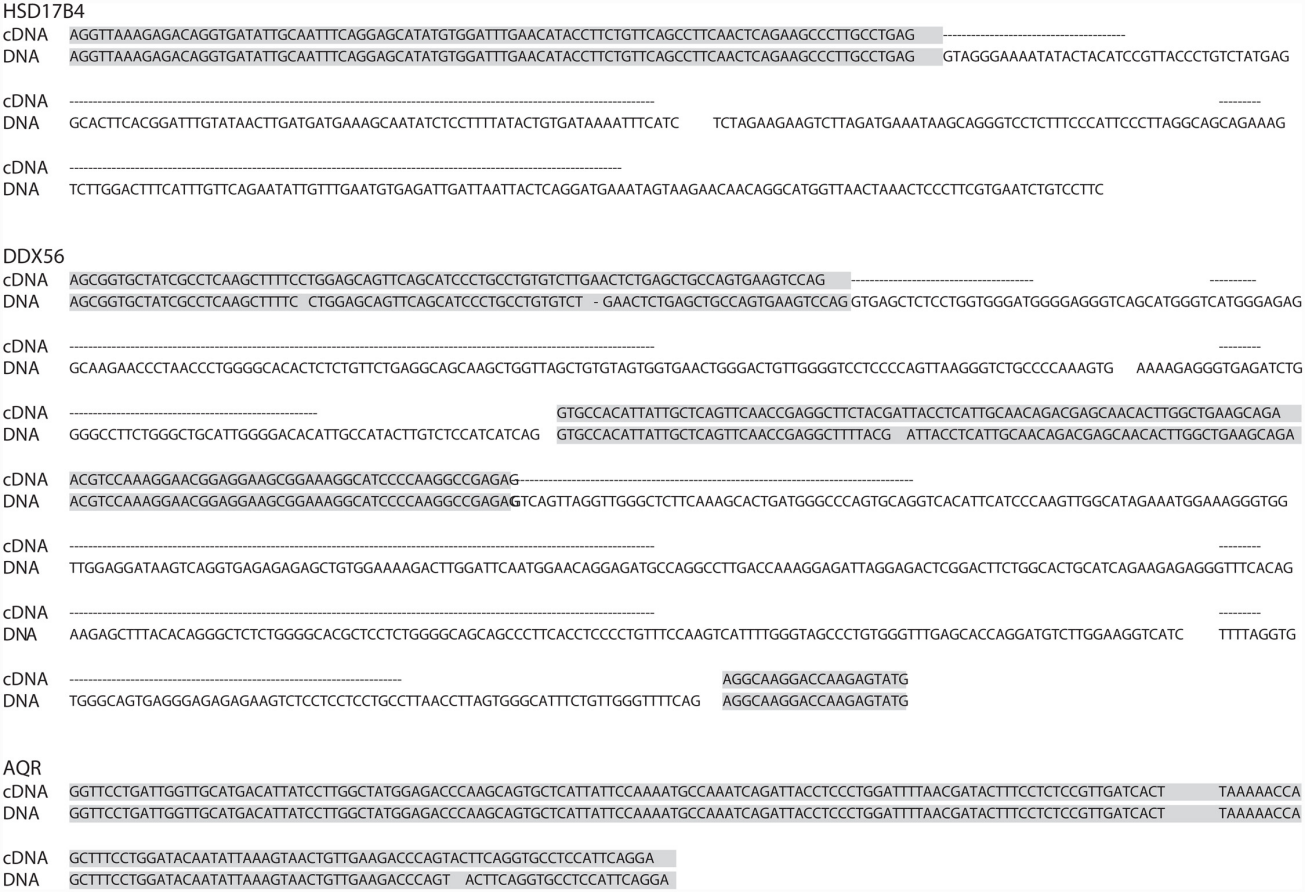
Sequencing of three representative chameleon genes amplified from DNA templates. Shown are alignments of sequences extracted from the RNA-seq (cDNA) and the corresponding genomic DNA sequence. Exons are shaded in gray. The genes analyzed were *HSD17B4*, comprising a single exon and its adjacent intron, *DDX56*, comprising two exons and their intervening sequence (intron) and *AQR*, corresponding to a single exon.

LEMONS analysis predicted the presence of 82,794 exon-exon junctions in 11,812 non-redundant transcripts with high similarity (e value < 10^−5^) to the human protein database. Simultaneous use of the human and five model organism genomes as references allowed LEMONS to predict 90,820 exon-exon junctions. This result represents an increase of 9.7% in the identified exon-exon junctions, relative to what was attained using the human reference database alone. To validate the predictions generated by LEMONS, we considered twenty different [N = 20] mRNA transcript sequences isolated from the chameleon transcriptome exhibiting high similarity to 20 human proteins associated with different functions and pathways (S4 Table). Predicted splice-junctions were used to design PCR primers, which, in turn, were employed to amplify gene sequences from chameleon DNA templates. Notably, some of the primer pairs were directed to the same predicted exon while others were directed to two adjacent exons, thus leading to expected amplification of only exonic or exon-intron-exon sequences, respectively. All of the designed fragments were successfully PCR amplified. Sequencing of 19 of 20 of the gene products revealed either exons and their intervening sequence, or a single exon (when both primers were designed from sequence within a given predicted exon) (Fig 5 and S4 Fig).

## Discussion

LEMONS enables the accurate identification of splice-junctions in multiple protein-coding transcripts. This approach, which analyzes RNA sequences independent of the corresponding genomic reference, is important for future genomic studies of most metazoa, a group in which only a small subset has been subjected to whole genome sequencing to date. Moreover, since RNA-seq can be *de novo* assembled without prior knowledge of genome organization, LEMONS represents a major advance in two main aspects. First, since LEMONS identifies splice-junctions in mRNA sequences alone, it allows the use of transcriptome data to predict gene architecture in under-studied species, thus extending the accessibility of many loci for subsequent functional analysis and allowing for the study of population variation involving these loci in multiple DNA samples. Second, LEMONS adds another dimension to the assessment of the functional potential of mutations solely utilizing RNA sequences. Specifically, once splice-junctions have been predicted and verified, the functionality of genetic variants within these sites can be evaluated, including those potentially affecting alternative splicing. Finally, LEMONS allows for the simultaneous use of multiple reference genomes. In addition, LEMONS allows users flexibility through a Graphical User Interface that lets them generate their own reference genomes from publicly available genomes and annotation data. Taken together, LEMONS could be particularly beneficial for ecologists and evolutionists investigating under-studied vertebrates. Here, LEMONS could accurately predict exon sequences to be used in the design of primers for PCR amplification and subsequent analysis of many genes that until now were practically inaccessible.

The logic that underlies LEMONS was previously used in CEPiNS, developed by Hasan and Wheat (17). However, unlike LEMONS, the accuracy of predictions made by CEPiNS was not estimated, and was not experimentally verified. Moreover, CEPiNS utilizes only one reference genome and does not include a motif search. These missing features are embedded in LEMONS, increased its precision and reducing its false discovery rate. Furthermore, our thorough comparison indicated that LEMONS outperformed CEPiNS in all parameters tested. Finally, the accuracy of LEMONS was thoroughly analyzed and the predictions made were verified experimentally. Hence, LEMONS is the only available experimentally validated tool that efficiently predicts splice-junctions regardless of the availability of a reference genome.

Using LEMONS, we successfully amplified and sequenced exons and introns in several genes found within the chameleon transcriptome (Fig 5 and S4 Fig). The ability to sequence non-coding DNA regions is especially attractive for phylogenetic and molecular ecology or evolutionary studies, since such sequences are less susceptible to selective pressure, and could thus accumulate more mutations and assist in calculating genomic mutation rates.

In summary, our approach bridges the gap between *de novo* assembled RNA-seq data and DNA-based studies. By relying on the evolutionary conservation of exon/intron structure, and incorporating a splice site recognition motif search, LEMONS effectively identified splice-junctions in sequences derived from *de novo* assembled RNA-seq data while avoiding the need for a reference genomic sequence. This allows the use of DNA samples as templates in downstream analysis, either in population genetics or phylogenetic studies, especially in non-model and under-studied organisms, corresponding to the vast majority of living vertebrates. Finally, as the cost of oligonucleotide synthesis drops, LEMONS will facilitate the design of custom-made probes for exome capture in vertebrates lacking a reference genome.

## Supporting Information

S1 Fig LEMONS similarity (A), sensitivity (B) and precision (C) as determined in an assessment of 41 vertebrate genomes. Comparison of LEMONS performance, plotted for 41 vertebrates. The nomenclature used is as in the legend to Fig 2. For an explanation of ‘Annotation Quality’, see Materials and Methods. Species: 1 –*Pan troglodytes*, 2 –*Gorilla gorilla gorilla*, 3 –*Pongo pygmaeus abelii*, 4 –*Callithrix jacchus*, 5 –*Tarsius syrichta*, 6 –*Microcebus murinus*, 7 –*Otolemur garnettii*, 8 –*Tupaia belangeri*, 9 –*Rattus norvegicus*, 10 –*Dipodomys ordii*, 11 –*Cavia porcellus*, 12 –*Oryctolagus cuniculus*, 13 –*Spermophilus tridecemlineatus*, 14 –*Ochotona princeps*, 15 –*Sus scrofa*, 16 –*Ovis aries*, 17 –*Equus caballus*, 18 –*Felis catus*, 19 –*Mustela putorius furo*, 20 –*Canis lupus familiaris*, 21 –*Ailuropoda melanoleuca*, 22 –*Myotis lucifugus*, 23 –*Pteropus vampyrus*, 24 –*Erinaceus europaeus*, 25 –*Sorex araneus*, 26 –*Loxodonta africana*, 27 –*Procavia capensis*, 28 –*Dasypus novemcinctus*, 29 –*Choloepus hoffmanni*, 30 –*Monodelphis domestica*, 31 –*Sarcophilus harrisii*, 32 –*Ornithorhynchus anatinus*, 33 –*Meleagris gallopavo*, 34 –*Taeniopygia guttata*, 35 –*Latimeria chalumnae*, 36—*Tetraodon nigroviridis*, 37 –*Takifugu Rubripes*, 38 –*Gasterosteus aculeatus*, 39 –*Oryzias latipes*, 40 –*Gadus morhua*, 41 –*Petromyzon marinus*
(DOCX)

S2 Fig Determining default values for motif lengths and minimal exon length in LEMONS. (A-E) Motif lengths of 1–5 bp (lines), in combination with a minimal exon length of 1–20 bp (X-axis), were tested using the human reference database on five model organisms (indicated). The Y-axis represents the precision gain to sensitivity loss ratio, relative to when the motif search feature was excluded. Precision = TP/(TP+FP), Sensitivity = TP/(TP+FN).
(DOCX)

S3 Fig Determining the LEMONS default e-value. Comparison of three different e-values. Use of an e value of < 1.0 X 10^−5^ allowed LEMONS to analyzed more orthologous sequences while showing almost no, if any, difference between sensitivity and precision. (A) similarity, (B) sensitivity and (C) precision.
(DOCX)

S4 Fig Sequencing of nine chameleon genes amplified from DNA templates. Shown are alignments of sequences extracted from the RNA-seq (cDNA) and the corresponding genomic DNA sequence. Exons are shaded in gray. *SDHC*, *POLRMT and ACAD9* each correspond to single exon and its adjacent intron, *POLE2*, *LARS*, *RBM5* and *ETFA* each correspond to two exons and their intervening sequence (intron), *ARHGEF*, *ANKRD11*, *GLN1*, *VPS11*, *MARS2*, *TCIRG1*, *TAP1* and *C1QBP* each correspond to a single exon and *MRPL30a* corresponds to a single intron.
(PDF)

S1 Table Primer sequences used for PCR amplification of chameleon genes. (DOCX)

S2 Table PCR reactions and amplification conditions. (DOCX)

S3 Table Absolute number of transcripts and splice-junctions. The data presented are taken from Fig 2D. **Transcripts:** Number of sequences that served as input for LEMONS. TP: Number of splice-junctions correctly identified by LEMONS. TP+FN: Total numbers of true splice-junctions in each species within the human orthologous sequences. TP+FP: Number of splice-junctions predicted by LEMONS. **Total Length**: Number of bases that served as input for LEMONS. **Total Analyzed:** Number of bases that were analyzed (i.e., the length of the orthologs which were analyzed).
(DOCX)

S4 Table Transcripts analyzed in the Mediterranean chameleon and their respective human protein accession numbers (Genbank). (DOCX)
