# Supplementary material for: LEMONS – A Tool for the Identification of Splice Junctions in Transcriptomes of Organisms Lacking Reference Genomes
Source: PLoS One. 2015 Nov 25;10(11):e0143329. doi: 10.1371/journal.pone.0143329 (PMC4659627; doi:10.1371/journal.pone.0143329)
Supplement: S2 Fig — (A-E) Motif lengths of 1–5 bp (lines), in combination with a minimal exon length of 1–20 bp (X-axis), were tested using the human reference database on five model organisms (indicated). The Y-axis represents the precision gain to sensitivity loss ratio, relative to when the motif search feature was excluded. Precision = TP/(TP+FP), Sensitivity = TP/(TP+FN). (DOCX) [file pone.0143329.s002.docx]

A B

C D

E
