# Supplementary material for: LEMONS – A Tool for the Identification of Splice Junctions in Transcriptomes of Organisms Lacking Reference Genomes
Source: PLoS One. 2015 Nov 25;10(11):e0143329. doi: 10.1371/journal.pone.0143329 (PMC4659627; doi:10.1371/journal.pone.0143329)
Supplement: S3 Fig — Comparison of three different e-values. Use of an e value of < 1.0 X 10−5 allowed LEMONS to analyzed more orthologous sequences while showing almost no, if any, difference between sensitivity and precision. (A) similarity, (B) sensitivity and (C) precision. (DOCX) [file pone.0143329.s003.docx]

ABC

10^-50^ 10^-10^ 10^-5^

10^-50^ 10^-10^ 10^-5^

10^-50^ 10^-10^ 10^-5^
