# Supplementary material for: LEMONS – A Tool for the Identification of Splice Junctions in Transcriptomes of Organisms Lacking Reference Genomes
Source: PLoS One. 2015 Nov 25;10(11):e0143329. doi: 10.1371/journal.pone.0143329 (PMC4659627; doi:10.1371/journal.pone.0143329)
Supplement: S4 Fig — Shown are alignments of sequences extracted from the RNA-seq (cDNA) and the corresponding genomic DNA sequence. Exons are shaded in gray. SDHC, POLRMT and ACAD9 each correspond to single exon and its adjacent intron, POLE2, LARS, RBM5 and ETFA each correspond to two exons and their intervening sequence (intron), ARHGEF, ANKRD11, GLN1, VPS11, MARS2, TCIRG1, TAP1 and C1QBP each correspond to a single exon and MRPL30a corresponds to a single intron. (PDF) [file pone.0143329.s004.pdf]

ARHGEF

cDNA CTTTGCTCTTTGCTCGCTGTTTCATTCTCTCATCTTCCTGTTTCTTTGATTTGGACACTGACCCCTTTGTGTCTGACCCAGAAATACTTTTGTATGGACAACTTGAGGATTTCTCTAAGGGTAAATGTTTTGTT
DNA CTTTGCTCTTTGCTCGCTGTTTCATTCTCTCATCTTCCTGTTTCTTTGATTTGGACACTGACCCCTTTGTGTCTGACCCAGAAATACTTTTGTATGGACAACTTGAGGATTTCTCTAAGGGTAAATGTTTTGTT

cDNA CTGCTGTTCTTTCTTAGAACCACAGTAGGAAGGGTCTCCATGCCTGATTTTTCCAACTCCATCCTCAAACTCTCAGGGTCTTTTACTACCTGTGGTGATACCATAGACCCTTCCATGTCCTCTGCATCTATTTC
DNA CTGCTGTTCTTTCTTAGAACCACAGTAGGAAGGGTCTCCATGCCTGATTTTTCCAACTCCATCCTCAAACTCTCAGGGTCTTTTACTACCTGTGGTGATACCATAGACCCTTCCATGTCCTCTGCATCTATTTC

cDNA TGCATTTGCTTGCCACCACTGGGGTTCCTCAGAAACAGCCTGTTGCCTCTGTGGCTGTCTCTTAGTTGGTGGTGATGGGGGACCAAGCTGGAGAAAGTGAATTGGGTTTGTGAAAGCCAAAAGTGAAGGAGTCT
DNA TGCATTTGCTTGCCACCACTGGGGTTCCTCAGAAACAGCCTGTTGCCTCTGTGGCTGTCTCTTAGTTGGTGGTGATGGGGGACCAAGCTGGAGAAAGTGAATTGGGTTTGTGAAAGCCAAAAGTGAAGGAGTCT

cDNA CTCTTGAATTTCTCGACTTTGTAGCTTTGCAACTTCTCTTCGCTTCTACC
DNA CTCTTGAATTTCTCGACTTTGTAGCTTTGCAACTTCTCTTCGCTTCTACC

POLE2

cDNA AGCAGTCGATTTGTGTTTGTTCCTGGCTCTGAAGACCCAGGTCCAGGTTCCATATTTCCA--
DNA AGCAGTCGATTTGTGTTTGTTCCTGGCTCTGAAGACCCAGGTCCAGGTTCCATATTGCCAAGGTATGGTTGTTACCATTAAATACTCTTACATATTTCTAGAGAATATGCAAATGATTCATCTCTGATACATAT

cDNA ----------------AGACCCCCCCTTGGAGAAAATATCACTGAGGAATTCAGACAGCAGGTGCCTTATTCTGTTTTCACTACAAATCCTTGCA
DNA CACATTAATTTATTTTAGACCCCCCCTTGGAGAAAATATCACTGAGGAATTCAGACAGCAGGTGCCTTATTCTGTTTTCACTACAAATCCTTGCA

LARS

cDNA TCCATCATGAAAGCTTCCTGGCCGGTGCCTGGCCCTGTGGATGAAGTACTCCTCCGCTCGTCCCAGTACCTCACTGAGGTAGCCCACGATCTCCGCCTGCGCCTTAAGAACTACATGGCACCAGCAAAAGGGAA
DNA TCCATCATGAAAGCTTCCTGGCCGGTGCCTGGCCCTGTGGATGAAGTACTCCTCCGCTCGTCCCAGTACCTCACTGAGGTAGCCCACGATCTCCGCCTGCGCCTTAAGAACTACATGGCACCAGCAAAAGGGAA

cDNA G---
DNA GGTAAGCCCTTCTTTTAGTGATGGCAAACCTTTTAGAGACCGAATGCCCAAAACCAACCCCACCCCTCCCTCCTCCTGCTACCTGTCTCACCCTGCACCTTCTCTTGCCCTCGCCTCCTCCTGGGCAGGATTTC

cDNA --
DNA AGCCTATGGGACCTGCAGAGAGCCTCCCCTCTCACCTCCCTATTGGCAGAGGGAGAAGAGTGGGTGGGGAAGTGCCACTGCCATTGACCCCAGTGGGGTGGGGCCTGACCAGTGTGCCCCGTGTGCCCACAGAA

cDNA --
DNA AGGGCTCTGGGCCCCTCCTCTGGCATGCATGACATAGGTTCATTACAACTGCCTTATATTGTCTCCTCCTTCTCCCATCATATGCTTTAAAGACACCTGTCTTCTAAGAGAGATTTTCCTTTAATGTTTCTTTT

cDNA ----AGAAGGGCAGCAAGGGAGCGCCCCAGAAGCCATCTCACTGCACCATTTATGTGGCAAAAAACTACCCACCATGGCAGCATATCACATTATCTGTTCTGCGCACGCAAGCATTATC
DNA TTGTAGAAGGGCAGCAAGGGAGCGCCCCAGAAGCCATCTCACTGCACCATTTATGTGGCAAAAAACTACCCACCATGGCAGCATATCACATTATCTGTTCTGCGCACGCAAGCATTATC

ANKRD11

```
cDNA  GCCGTCGGTGTGTTTCTCTCGGTGCTTCTCCCTCTCTTCTTTCCATTTCTCTTTGTGCCGGTCTCTCTTCTTCTTCTCTTTGGTGGCGCTGTATGTTCTCTCCTTTTCCGCCTTCTTTGGAAGCTCTCGTTCAG
DNA   GCCGTCGGTGTGTTTCTCTCGGTGCTTCTCCCTCTCTTCTTTCCATTTCTCTTTGTGCCGGTCTCTCTTCTTCTTCTCTTTGGTGGCGCTGTATGTTCTCTCCTTTTCCGCCTTCTTTGGAAGCTCTCGTTCAG

cDNA  ACTCATTTTTGTCCTTCTTTTCTGAAAATGGGTACTCTGTGTTTCTGTCTGGTTCTTCTTCTGCCTCTGGTTTGGTACCATAGGAAATGCCAAAGCCTTCTCCGTCAGCCACAAACTCCTTTTCATACTGGGTT
DNA   ACTCATTTTTGTCCTTCTTTTCTGAAAATGGGTACTCTGTGTTTCTGTCTGGTTCTTCTTCTGCCTCTGGTTTGGTACCATAGGAAATGCCAAAGCCTTCTCCGTCAGCCACAAACTCCTTTTCATACTGGGTT

cDNA  GAATCCTTCCTGTCTTTAAACTCGTCTATTTTATCCTTCTTCTCTGCTTTGTCTTTCTTGGTTTTCTCTTTTTCGTGGCTCTTCTTTGATGAGGATGAGGAATGCCGGTGCCTCTCCTTTTCCTTGAGTTTATC
DNA   GAATCCTTCCTGTCTTTAAACTCGTCTATTTTATCCTTCTTCTCTGCTTTGTCTTTCTTGGTTTTCTCTTTTTCGTGGCTCTTCTTTGATGAGGATGAGGAATGCCGGTGCCTCTCCTTTTCCTTGAGTTTATC

cDNA  CGGCAAGCCAGGCAAAGGCAAAGACG
DNA   CGGCAAGCCAGGCAAAGGCAAAGACG
```

GLN1

```
cDNA  AGTGGACTTGAGCAGCTGGAAGGAGAAAATTGAGCGGGATGCTGCGATGGCCCAGCACACCCTCGAGGACTCGGAGGATTCTGAGGAAGACGACGAAAGTCCGGCGGTATTAGTAGAGCACCAAACTGATGTGG
DNA   AGTGGACTTGAGCAGCTGGAAGGAGAAAATTGAGCGGGATGCTGCGATGGCCCAGCACACCCTCGAGGACTCGGAGGATTCTGAGGAAGACGACGAAAGTCCGGCGGTATTAGTAGAGCACCAAACTGATGTGG

cDNA  CTATGGAGGCCGGGGGATTCACGCAGGAGA
DNA   CTATGGAGGCCGGGGGATTCACGCAGGAGA
```

RBM5

```
cDNA  ACCTGTTATTGTAGCTGCATCTGTGTCCAATGTTCCTGCTTGGTTCTGGTAAAACTGTTGATAATCCTGTGAACACT---------------------------------------------------------
DNA   ACCGGTTATGGAAGCTGCATCTGTGTCCAATGTTCTTGCTGGGTTCTGGTAAAACTGTTGATAATCCTGTGAACACTATGCAGAGAATAAAGTCAAATTTACACCAGTGATTTCAAAATTAACATTATGTGAGT

cDNA  --------------------------------------------------------------------------------------------------------------------------------------
DNA   CTTACAAGAAACGGGTAATCATAGGACAGCTATCATAGGACCACCAGACACATTTCCTTTAAGAGAGCATTTGACTAAAGGAGGACAACTATGAAAAAAACCCCGCTTAACAGGTACTTCTAAAACATGGGGAC

cDNA  --------------------------------------------------------------------------------------------------------------------------------------
DNA   TCTGTGGGTTCTGCCCCATCTGGCACAAAACCTTCCTTCTATAAACTTAACTACATGGACTAACATTAGGATCAAATCTATGCTCTTGTGCTTTAAGTGCCTAACTCACATAGTTTTAGCTACACTTTTGTGGT

cDNA  --------------------------------------------------------------------------------------------------------------------------------------
DNA   GTATCCTTTGGTTTTTCTTAAGCATTAGGAAGCTTTGTTCTGTCCTTCTGATAGCTTTCTAGTAACACATTTCAAATTTTTAAATGTCATTTGACTTAACTGTCCTGTTTTCTTTTTAGCACAACTATGTAATA

cDNA  --------------------------------------------------------------------------------------------------------------------------------------
DNA   TCCAGGCAAGTTAAATAACACAGCACCTCACAAATACAAACATTAATGAAATGAAACATATCACATCTCCATCTTCTTGGTTGCCTGTGACTTTAAGAAGCAACTTCAGAGGGTCAAAAGGTACTTCTAAACCA

cDNA  --------------------------------------------------------------------------------------------------------------------------------------
DNA   TGGAATGATGGGGAAACTTACCTGCTTTTTGGTAATTCATCACTCCTTCCTCCCACAGCACCCACCCTTGCATGTAATTAGTGTTCAGAAACACCTTTCAGTACTTCTAAAGAGTTAATGGAGAACAGAAAACT
```

```
cDNA  -------------------------------GAGAGTACTGGGAATAACCATCCTGCCCTAG
DNA   TTTTCACTTGTAGCACTATTCAGACCCACCTGAGAGTACTGGGAATAACCATCCTGCCCTAG
```

VPS11

```
cDNA  GGTGCGTTTGGTGTGCATGAGCATGTCACGCTGCAGGCCAGCCTCCAGAGCTGTGGCTGACTTGCTGGATGGAAGGTCAGTGAGAAGGGTGATCTTGTTAAA
DNA   GGTGCGTTTGGTGTGCATGAGCATGTCACGCTGCAGGCCAGCCTCCAGAGCTGTGGCTGACTTGCTGGATGGAAGGTCAGTGAGAAGGGTGATCTTGTTAAA
```

SDHC

```
cDNA  CTGTCTCCTCATGTGACCATCTATGA------------------------------------------------------------------------------------------------------------
DNA   CTGTCTCCTCATGTGACCATCTATGAGTAAGAGCAGTCTTCTCTGATGAAGGGGGAAATTTTTCCTATTTGTTTGAAATGCATTTGATTGAGGTCAAGATGTTTTACAATTTTTTAAAAACAAGTTCATTTCCT

cDNA  --------------------------------------------------------------------------------------------------------------------------------------
DNA   TCCTGCTCATCCTCTCAAAAAGCAGCATTAGTGGGGATTAATCCTGGCAACCTAGCAGACTGAAAGCAAGAAATTTCAAATACAGTAGATAAATCGGGACCATTTCGGTGGGAAAAGATCTTGGGAACATGAAA

cDNA  -----------------------------------------------------------
DNA   GGAGCCTCATTTGGGTGTCCTCTGAGTAGAGATTTTCCAGCAAAAATCCTTTCAATCCA
```

POLRMT

```
cDNA  TTGGGGCTTCCTATCTCCACCTACCATCGCTCTAAAACCATCCTGG----------------------------------------------------------------------------------------
DNA   TTGGGGCTTCCTATCTCCACCTACCATCGCTCTAAAACCATCCTGGTAAGCTGTGGATCACAACGGCTGGGTGGTGAGGTGGTGGTGGAGAGCAGAACGAGGCCATGTACAATAGCTAAGAGGTATCCTTTTCC

cDNA  ----------------------------
DNA   TTAGTCAGCCTTTTAGCATCTTCTCTCT
```

MARS2

```
cDNA  TCTCATGGCCCAGGGCGTGCCACGTTGTGGGCAAAGATATTCTCAAGTTCCATGCCATCTATTGGCCTGCTCTGCTGATGGCAGCAGGACTGGAGCCTCCGGAACAGATCTTGGTCCATTCTCACTGGACAGTG
DNA   TCTCATGGCCCAGGGCGTGCCACGTTGTGGGCAAAGATATTCTCAAGTTCCATGCCATCTATTGGCCTGCTCTGCTGATGGCAGCAGGACTGGAGCCTCCGGAACAGATCTTGGTCCATTCTCACTGGACAGTG

cDNA  GAAGGGCAGAAGATGTCCAAGAGCCTAGGCAACGTGGTGGACCCAGTCACCTGCTTCCAGCAGTACTCAGTGGATGGATTCCGGTATTTCCTACTGAGACAGGGTGTCCCTGAGTGGGACAACGACTACTACAG
DNA   GAAGGGCAGAAGATGTCCAAGAGCCTAGGCAACGTGGTGGACCCAGTCACCTGCTTCCAGCAGTACTCAGTGGATGGATTCCGGTATTTCCTACTGAGACAGGGTGTCCCTGAGTGGGACAACGACTACTACAG

cDNA  CCGGAAGGCCATCAACCTGATCAATTCGGAGCTGGCTGATGCCCTTGGAGGACTCCTCAACCGGTGTACGGCCTCCAGTCTGAACCCCAGTGGGATCTTCCCTTGCTTTTCGGAGGAATGCTTTCCCCAGGATA
DNA   CCGGAAGGCCATCAACCTGATCAATTCGGAGCTGGCTGATGCCCTTGGAGGACTCCTCAACCGGTGTACGGCCTCCAGTCTGAACCCCAGTGGGATCTTCCCTTGCTTTTCGGAGGAATGCTTTCCCCAGGATA

cDNA  TGAATGCTGAAAGGGTAAGAGGATCTGCTTGGGCCTCAGCAGATGATTACATGTTGGTGTCATCAGTGAAAGACTTGCCCATAAAAGTGAGCCGTTCGTTTGAAGAATTTTGCATCTACAAGGCTTTGGAGGCC
DNA   TGAATGCTGAAAGGGTAAGAGGATCTGCTTGGGCCTCAGCAGATGATTACATGTTGGTGTCATCAGTGAAAGACTTGCCCATAAAAGTGAGCCGTTCGTTTGAAGAATTTTGCATCTACAAGGCTTTGGAGGCC
```

```
cDNA  ATCTCAGATTGCGTGAGGCAGACCAACAGCTTCATCCAGAGACATGCTCCTTGGAAACTCAACCTCGAGGAGCCAGCACAGAAGCGCTGGCAGGACACCATCATCCACGTCACTCTGGAATGTCTCCGGGT
DNA   ATCTCAGATTGCGTGAGGCAGACCAACAGCTTCATCCAGAGACATGCTCCTTGGAAACTCAACCTCGAGGAGCCAGCACAGAAGCGCTGGCAGGACACCATCATCCACGTCACTCTGGAATGTCTCCGGGT
```

MRPL30

```
cDNA  --------------------------------------------------------------------------------------------------------------------------------------
DNA   GGAAGGGAAGGATCGGAAAGGGGTCCCCGGGTGGAATGTGGCCGTGGGTTTAGAAAACAGCCCTCTATGTATAAGAGAGGGAGGGTTGTAATGTTCTAATATGTTTTCTTTAACGTTGATGGTGCCTTTTTACG

cDNA  --------------------------------------------------------------------------------------------------------------------------------------
DNA   GCTTCTATCCGTGGGCTTTATTTATCTTTTAGTTGAAAATGTTGTAAGCCGCCCAGAGTCGCGTGTTGATTGGGCGGCAATACAAAGCGAATTAAATAAATAAATTTTGTCAAAAGTTAAAAAGCTAAGGTTCG

cDNA  --------------------------------------------------------------------------------------------------------------------------------------
DNA   AAAGCAAGAAATTGCAAATAGACAAATAGGTACCGCTTCAGTGGGAAGGTAACAGCGTTCTGTGTTCTCCATTGTCTATTCCTGCCTGACACACGATCACAGAAACATCTGCAGAAGCAGACGGTGATAGTTCT

cDNA  ------------------------------------------------------------------------------------------------
DNA   TCGGCTTTGAAACAGAGATGATCACTGAACCCTAGAGTCAGATACGACTGGCTTTTGAGCAAGGAAAACCTTTACTTTACCTTTATAAGAAGCTTA
```

ACAD9

```
cDNA  CCATCCCAGTCTACAA----------------------------------------------------------------------------------------------------------------------
DNA   CCATCCCAGTCTACAAGTAAGTAGCTAACACAAATCTAGGAGGTATGCCTGGGTATCTTCTTTCTTGTACAAGTATGGATGTTTGCAGGCTGTCTAGTAAGTTGATTCTGGTATTCTTTATTGTTCACACCAAT

cDNA  --------------------------------------------------------------------------------------------------------------------------------------
DNA   CTAATGACATGGAACTTGATCTAGAGCTGCTAGAGGTTTTCTTCACTGATACTTGCAAGATGAAGCAACATATGGGTTTAACTAGGTACAGTTGTAATGTTAATAAAATGTTGAATGTTGCTCTTTTTCTAAAA

cDNA  --------------------------------------------------------------------------------------------------------------------------------------
DNA   TTGTTAACTTTGCTGGCCGGTTCTTAAATGTACATATTTGCACGCACATAATGTGTTGAAATAAGAGGACCTGCTTCTTGTTTGAAATGGTTGCCTTTCAAAAATAAGGCTGAATCCATAACGTAGAGTATTAT

cDNA  --------------------------------------------------------------------------------------------------------------------------------------
DNA   CACCACCCCGTATCTGTAGAGGATACATTCTAGGATTTTACACGTATTCTTGAAACCACGGAAAACAGCAAACCCTGTAATAAGAACATATACCTGAGTATGGCAGTTCAGTTGCTCAATATTTTTAAAACTGC

cDNA  ----------------------------------
DNA   ATATATCAGTTGTATGTGGGTAATTGAGAATGAA
```

TCIRG1

```
cDNA  AGGTGGAGTAGCCCTTGTACCCATCTTCGCTTTCTTTGCGGTACTGACCGTAGCCATCTTGCTAGTGATGGAGGGTCTGTCAGCTTTTTTGCATGCTTTACG
DNA   AGGTGGAGTAGCCCTTGTACCCATCTTCGCTTTCTTTGCGGTACTGACCGTAGCCATCTTGCTAGTGATGGAGGGTCTGTCAGCTTTTTTGCATGCTTTACG
```

TAP1

```
cDNA  CTAGGGAAGGTGAGCAGTGGTGAACTTGTCATGTTCGTCCTCTACGAGATGCAGTTTTCCAC
DNA   CTAGGGAAGGTGAGCAGTGGTGAACTTGTCATGTTCGTCCTCTACGAGATGCAGTTTTCCAC
```

C1QBP

```
cDNA  GAATTCCTGACAGATGAAATCAAGGAAGAGAAGATAATCCAGAAACACAAAGCCCTCCCTAAA
DNA   GAATTCCTGACAGATGAAATCAAGGAAGAGAAGATAATCCAGAAACACAAAGCCCTCCCTAAA
```

ETFA

```
cDNA  CTCAACATGATGCCTACAAAGGACTACTGCCTG----------------------------------------------------------------------------------------------------A
DNA   CTCAACATGATGCCTACAAAGGACTACTGCCTGGTGAGGCTTATTCTCAAACACAGCTTCGCATTAAAGTTTCCTGCAGTCAGCTTTGTGCTAGAATAGATAAGGTGCCCTGACTCGTCTGTCTCGTCCCCAGA
cDNA  GGAGCTGACTCC
DNA   GGAGCTGACTCC
```
