# Supplementary material for: LEMONS – A Tool for the Identification of Splice Junctions in Transcriptomes of Organisms Lacking Reference Genomes
Source: PLoS One. 2015 Nov 25;10(11):e0143329. doi: 10.1371/journal.pone.0143329 (PMC4659627; doi:10.1371/journal.pone.0143329)
Supplement: S1 Table — (DOCX) [file pone.0143329.s005.docx]

**S1 Table:** Primer sequences used for PCR amplification of chameleon genes.

| **Gene** | **Primers (F-forward; R-reverse)** | **Primer sequences** |
| --- | --- | --- |
| *DDX56* | F | CGGGTCCTGGCTCACTTGCAAG |
|  | R* | CAAAGTTCAGGACAGCCGAGAC |
| *ANKRD11* | F* | GGCTGATGGTTTGAGAGCAGTC |
|  | R | GTCTTTGCCTTTGCCTGGCTTG |
| *POLE2* | F* | AGCAGTCGATTTGTGTTTGTTC |
|  | R* | TGCAAGGATTTGTAGTGAAAAC |
| *GLN1* | F | GTGGACTTGAGCAGCTGGAAGG |
|  | R* | TACGCAGCCTATGGTTAAAATC |
| *AQR* | F* | GCTGTTTTGGAAACAATAAGG |
|  | R | CCTGAATGGAGGCACCTGAAG |
| *LARS* | F* | TGTCTGATAATGCTTGCGCAG |
|  | R* | AAGCCTCACTCCATCATGAAAG |
| *HSD17B4* | F* | CAGCTGATTTCCATCTTCTTTG |
|  | R | AAGGTTAAAGAGACAGGTGA |
| *KIAA0020* | F | ACACAAAATAATTGCACCACG |
|  | R | CTTCACATCGCAGAACACCCAG |
| *ARHGEF5* | F* | CTTCTAATACACTGTCTGTGGG |
|  | R | GTAGAAGCGAAGAGAAGTTGC |
| *RBM5* | F | ACCTGTTATTGTAGCTGCATC |
|  | R* | CCACAAAGTGGAGAAGGTGGTG |
| *VPS11* | F | GGTGCGTTTGGTGTGCATG |
|  | R* | CTGAAGTGTTCCAACGACAGC |
| *POLRMT* | F* | GCCCGTTTCATTGCCAAATCTG |
|  | R | GTGGTATCGTGGCTCCATTTG |
| *SDHC* | F* | CCAATGGGAACAACAGCAAAAG |
|  | R | CATTGCAATTCCTGTACCCCG |
| *MARS2* | F* | CCAGAGGATGCATCACAAACC |
|  | R | CACCGGTTGTAGCAGGATCCC |
| *MRPL30* | F* | TGCGGTAGGGGGCGATTGCTC |
|  | R | ATTCAGGAACTCTTGCTTTTG |
| *ACAD9* | F* | TTTGCATGATACCTTTGGTCG |
|  | R | GCCAAACCTCCTTAGCAAGCC |
| *TCIRG1* | F* | CTCTCAGAGGTGCTTTGGAG |
|  | R | CGTAAAGCATGCAAAAAAGC |
| *TAP1* | F* | CTGGACTAGCCCTAAAAGTGG |
|  | R | GTGGAAAACTGCATCTCGTAG |
| *C1QBP (P32)* | F | GAATTCCTGACAGATGAAATC |
|  | R* | GCTACTTTCCGGGTAAGCTTG |
| *ETFA* | F | CTCAACATGATGCCTACAAAG |
|  | R* | GCAGATGCTCCGACACAGATG |

* = used for Sanger sequencing.
