# Supplementary material for: LEMONS – A Tool for the Identification of Splice Junctions in Transcriptomes of Organisms Lacking Reference Genomes
Source: PLoS One. 2015 Nov 25;10(11):e0143329. doi: 10.1371/journal.pone.0143329 (PMC4659627; doi:10.1371/journal.pone.0143329)
Supplement: S2 Table — (DOCX) [file pone.0143329.s006.docx]

**S2 Table.** PCR reactions and amplification conditions

| **Gene** | **Reaction mix** | **PCR conditions** |
| --- | --- | --- |
| *DDX56, POLRMT, SDHC and KIAA0020* | 5 pmol of forward and reverse primers each, 1.25 units Phusion polymerase (Thermo), 1 x reaction buffer (Thermo), 0.2 mM dNTP mix, and ~50 ng DNA as template. | 98°C for 5 minutes, followed by 35 cycles including denaturation (98°C, 30 sec), annealing (66°C, 20 sec) and elongation (72°C; 120 seconds for DDX, POLRMT and SDHC, 240 seconds for KIAA0020). The cycles were followed by a final extension step (72°C, 7 min) |
| *ANKRD11, POLE2, AQR, RBM5, HSD17B4, LARS, GLN1, VPS11, ARHGEF5, MARS2, MRPL30, ACAD9, TCIRG1, TAP1, C1QBP (P32) and ETFA* | 5 pmol of forward and reverse primers (each), 1.25 units Taq polymerase (Bio-Lab), 1 x reaction buffer (Bio-Lab), 2 mM MgCl2 and 0.2mM dNTP mix, as well as ~50 ng DNA as template. | 94°C for 5 minutes, followed by 35 cycles of denaturation (94°C, 30 sec), annealing (60°C for *MRPL30*; 66°C for *TAP1* and *ETFA*; 58°C, 30 sec for all the rest) and elongation (72°C, 15 sec for *TAP1*, *TCIRG1*, *C1QBP*, and *ETFA*; 120 sec for *MRPL30*, 60 sec for all the rest). The cycles were followed by a final extension step (72°C, 7 min). |
