# Supplementary material for: LEMONS – A Tool for the Identification of Splice Junctions in Transcriptomes of Organisms Lacking Reference Genomes
Source: PLoS One. 2015 Nov 25;10(11):e0143329. doi: 10.1371/journal.pone.0143329 (PMC4659627; doi:10.1371/journal.pone.0143329)
Supplement: S3 Table — The data presented are taken from Fig 2D. Transcripts: Number of sequences that served as input for LEMONS. TP: Number of splice-junctions correctly identified by LEMONS. TP+FN: Total numbers of true splice-junctions in each species within the human orthologous sequences. TP+FP: Number of splice-junctions predicted by LEMONS. Total Length: Number of bases that served as input for LEMONS. Total Analyzed: Number of bases that were analyzed (i.e., the length of the orthologs which were analyzed). (DOCX) [file pone.0143329.s007.docx]

**S3 Table: Absolute numbers of transcripts and splice junctions.** The presented data correspond to Figure 2D. **Transcripts:** number of sequences that were used as input for LEMONS.TP: number of correctly identified splice junctions by LEMONS.TP+FN: Total numbers of true splice junctions in each species within the human orthologous sequences**.** TP+FP: number of predicted splice junctions by LEMONS. **Total Length**: number of bases that were used as input for LEMONS. **Total Analyzed:** number of bases that were actually analyzed (the length of the orthologs which were analyzed).

|  | ***M. musculus*** | ***G. gallus*** | ***A. carolinensis*** | ***X. tropicalis*** | ***D. rerio*** |
| --- | --- | --- | --- | --- | --- |
| **Transcripts** | 41,430 | 16,165 | 19,090 | 22,578 | 36,820 |
| **TP** | 281,748 | 128,335 | 130,122 | 156,447 | 218,444 |
| **TP+FN** | 290,554 | 137,960 | 147,768 | 190,813 | 248,720 |
| **TP+FP** | 299,507 | 139,965 | 148,547 | 185,926 | 254,744 |
| **Total Length (bp)** | 58,429,034 | 27,095,233 | 30,422,777 | 36,503,596 | 56,239,887 |
| **Total Analyzed (bp)** | 52,009,260 | 23,227,230 | 25,860,978 | 30,923,385 | 43,061,895 |
