# Supplementary material for: LEMONS – A Tool for the Identification of Splice Junctions in Transcriptomes of Organisms Lacking Reference Genomes
Source: PLoS One. 2015 Nov 25;10(11):e0143329. doi: 10.1371/journal.pone.0143329 (PMC4659627; doi:10.1371/journal.pone.0143329)
Supplement: S4 Table — (DOCX) [file pone.0143329.s008.docx]

**S4 Table. Transcripts analyzed in the Mediterranean chameleon and their respective human proteins accession numbers (GenBank).**

| **Gene (Transcript)** | **Accession number** | **Gene (Transcript)** | **Accession number** |
| --- | --- | --- | --- |
| *DDX56* | NP_061955 | *ARHGEF5* | NP_005426 |
| *ANKRD11* | NP_001243112 | *POLMRT* | NP_005026 |
| *POLE2* | NP_002683 | *SDHC* | NP_002992 |
| *AQR* | NP_055506 | *MARS2* | NP_612404 |
| *RBM5* | NP_005769 | *MRPL30* | NP_660213 |
| *HSD17B4* | NP_000405 | *ACAD9* | NP_054768 |
| *LARS* | NP_064502 | *TCIRG1* | NP_006010 |
| *KIAA0020* | NP_055693 | *TAP1* | NP_000584 |
| *GLN1* | NP_005266 | *C1QBP (P32)* | NP_001203 |
| *VPS11* | NP_068375 | *ETFA* | NP_000117 |
